# Multivariate Survival Analysis of a Comprehensive Clinical Trial of Rapamycin in Amyotrophic Lateral Sclerosis Explores Prognostic Factors and Survival Patterns in C9orf72 Carriers

**DOI:** 10.1111/ene.70756

**Published:** 2026-09-19

**Authors:** Alex De Nardi, Alessio Paris, Mario Lauria, Ilaria Martinelli, Elisabetta Zucchi, Cecilia Simonini, Jessica Mandrioli, Luca Marchetti

**Affiliations:** ^1^ Fondazione The Microsoft Research ‐ University of Trento Centre for Computational and Systems Biology (COSBI) Rovereto Italy; ^2^ Department of Mathematics University of Trento Trento Italy; ^3^ Department of Neurosciences, Ospedale Civile Baggiovara Azienda Ospedaliero Universitaria di Modena Modena Italy; ^4^ Department of Biomedical, Metabolic and Neural Sciences University of Modena and Reggio Emilia Modena Italy; ^5^ Department of Cellular, Computational and Integrative Biology (CIBIO) University of Trento Trento Italy

**Keywords:** amyotrophic lateral sclerosis, biomarkers, C9orf72, rapamycin, survival analysis

## Abstract

**Background:**

Amyotrophic lateral sclerosis (ALS) is a progressive neurodegenerative disease marked by considerable variability in survival times. This retrospective study aims to evaluate the prognostic value of a broad range of variables by conducting a comprehensive survival analysis on longitudinal data from RAP‐ALS, a clinical trial investigating the effects of rapamycin in ALS patients (*n* = 63).

**Methods:**

Covariates were classified as risk or protective factors according to their hazard ratios. Regularized Cox regression was utilized to select the best‐performing multivariate models in cross‐validation. Longitudinal measures were incorporated by modeling covariates as time‐dependent. Survival times of treated C9orf72 mutation carriers (*n* = 6) were further investigated through log‐rank tests and restricted mean survival time analysis.

**Results:**

Univariate analyses confirmed several previously established prognostic factors. Multivariate regularized Cox models incorporated neurofilaments, creatinine, clinical scores, and markers of immune activation. Moreover, the inclusion of time‐varying covariates allowed us to investigate late‐stage risk factors, such as the neutrophil‐to‐lymphocyte ratio. Additionally, the analysis indicated a protective effect in treated C9orf72 mutation carriers (log‐rank test, *p* = 0.026), which was confirmed after comparing this treatment subgroup with an independent C9orf72+ cohort (*n* = 40; RMST test, *p* = 0.04).

**Conclusions:**

The survival analysis confirmed the role of previously identified prognostic factors, while suggesting that high‐performing multivariate models should integrate multiple ALS pathological hallmarks. Moreover, the observed longer survival among C9orf72 mutation carriers contrasts with prior reports. Due to the small sample size and potential confounding factors, the observed benefits in treated C9orf72 patients should be considered exploratory, supporting evaluation in a larger, genetically stratified trial.

## Graphical abstract

We performed survival analysis on data from RAP‐ALS, a clinical trial investigating the effects of rapamycin on amyotrophic lateral sclerosis patients. We confirmed the prognostic role of factors such as neurofilament, ALSFRS‐R, and creatinine, and we explored late‐stage risk factors (e.g., neutrophil‐to‐lymphocyte ratio) by modeling time‐varying covariates. We observed a protective effect in C9orf72 mutation carriers, which was confirmed in an independent cohort, suggesting a potential treatment effect that warrants further investigation.

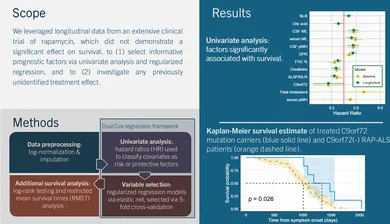

## 1 Introduction

Amyotrophic lateral sclerosis (ALS)—a progressive, fatal neurodegenerative disease affecting lower and upper motor neurons—is expected to impose an increasingly significant burden on public health, with incidence estimated to rise by 69% in the upcoming years [1]. Given the poor prognosis associated with ALS, there is a critical need for effective disease‐modifying therapies. Currently, the most impactful drug, tofersen, offers improved outcomes only for patients with a SOD1 mutation [2], while riluzole, the only other drug approved in Europe, can extend survival by only a few months [3].

In the context of exploring potentially effective drugs for ALS, the recent RAP‐ALS trial [4] evaluated the effect of rapamycin, a drug that targets both protein misfolding and immune system dysfunction, two well‐established pillars of ALS pathogenesis. No difference in survival or disease progression (as measured by the ALS Functional Rating Scale Revised—ALSFRS‐R score) was observed between randomization arms. Nonetheless, the study encompasses a wide range of measured covariates, allowing for an in‐depth assessment of the prognostic value of numerous measures commonly used in clinical practice.

Given the substantial variability in survival times following an ALS diagnosis [5], accurately evaluating a patient's status and prognosis is crucial for ensuring proper randomization and for effectively monitoring treatment efficacy in clinical trials. Although clinical scales can aid this process, they may be affected by varying degrees of subjectivity [6, 7]. Soluble biomarkers, in contrast, yield more robust and objective data, while being able to anticipate observable clinical changes due to their proximity to the underlying disease processes [8]. In neurodegenerative diseases, soluble biomarkers have been successfully employed for early diagnosis, patient stratification, and assessment of drug efficacy [9]. Combining biological and phenotypic measures in a multivariable prognostic model can provide personalized survival predictions for ALS patients, and machine learning methods can further support this task [10, 11].

The present study aimed to perform a retrospective study on longitudinal data collected in the context of the previously mentioned RAP‐ALS clinical trial [4]. Our first objective was to extract the most informative factors for predicting tracheostomy‐free survival in the trial's cohort. We examined the association between survival and both baseline data (i.e., the earliest available measurements) and time‐varying covariates, incorporating all available measures. Given the extensive range of variables, we employed variable selection methods to identify those with the greatest prognostic value and elucidate the relative contributions of different biological processes to survival outcomes. Our second objective was to investigate potential concealed treatment effects that may have remained undetected in previous analyses, including survival patterns in C9orf72 mutation carriers. Importantly, the C9orf72 subgroup analysis was not pre‐specified and is therefore post hoc and exploratory. Due to unbalanced randomization of this subgroup, these findings should be interpreted exclusively as hypothesis‐generating and may serve to inform the design of future clinical trials.

## 2 Methods

### 2.1 Data Collection

Data have been collected in the context of RAP‐ALS, a multicenter, randomized, double‐blind clinical trial [4]. The study cohort included 63 patients, collected from 7 Italian ALS referral centers between 2017 and 2020. Data were collected at up to 5 time points (0, 8, 18, 30, 54 weeks), depending on individual dropout times. Patients were stratified according to the disease progression rate (defined as the monthly decrease in the ALSFRS‐R score) and were subsequently randomized to placebo, 1 mg/m^2^/day rapamycin, and 2 mg/m^2^/day rapamycin. Treatment was administered for 18 weeks.

Patients with a known SOD1 mutation, with symptom onset earlier than 18 months before screening or not treated with riluzole, were excluded [4].

The primary outcome, defined as an increase of regulatory T cells (Tregs) of at least 30% with respect to baseline at treatment end (18 weeks), was not attained. Among secondary biological outcome measures, changes in B and T cell subpopulations, monocytes, and IL‐18 were observed. Moreover, no significant difference in survival times or ALSFRS‐R slope was observed between randomization arms.

### 2.2 Clinical Features

The cohort of patients is characterized by a comprehensive set of clinical and biological features. Demographic data included age, weight, body mass index (BMI), and sex. Disease‐specific information included the time of symptom onset, the site of onset, and the presence of C9orf72 hexanucleotide repeat expansions (HRE).

Functional assessments were performed using the ALSFRS‐R and forced vital capacity (FVC). The disease progression rate (DPR) was calculated as the monthly decline in ALSFRS‐R score from symptom onset.

Biomarker collection included the quantification of neurofilament light and heavy concentrations in both cerebrospinal fluid (CSF) and serum samples; up to two CSF measures per patient are available (weeks 0 and 18). Clinical laboratory measures encompassed common‐practice metabolites, inflammation markers, immune cell counts, and cytofluorimetry data.

The full list of available variables can be found in Table S1.

### 2.3 Control C9orf72 Data

Additional data from ALS patients with a C9orf72 mutation (CTRL‐C9) that weren't part of the RAP‐ALS trial were utilized in the analysis. These data consist of 53 patients from the Emilia‐Romagna ALS registry (ERRALS) who have been diagnosed with ALS between 2008 and 2023 [12, 13].

### 2.4 Data Preprocessing

The primary outcome variable in this study was survival time, defined as the number of days until either death or invasive ventilation (tracheostomy‐free survival). To assess the impact of covariates, survival time was calculated from the sampling date, while overall survival comparisons between patient classes used the estimated day of symptom onset as the starting point. Each predictor variable has been standardized to have a mean of 0 and a variance of 1. Biological variables underwent log‐normalization. The ALSFRS‐R scores were subjected to a logit transformation. To address missing data in the patient time series, we applied multiple imputation (50 imputed datasets) [14]. For CSF biomarkers, which were only measured at weeks 0 and 18 by study design, we imputed each patient's last observed value across subsequent time points. This was supported by the relatively low intra‐individual variance of CSF measures compared with their inter‐individual variability (Figure S1). The fraction of missing values for each variable is shown in Table S1.

Data are summarized using medians and interquartile ranges (IQR).

### 2.5 Statistical Analysis

The effect of variables on survival was assessed employing two distinct Cox proportional hazards modeling strategies [15] to address different assumptions regarding temporal covariate behavior:

– The ‘baseline Cox model’ utilizes *time‐fixed covariates*, derived from measurements at time 0, and assumes that the relative hazard between individuals remains constant throughout follow‐up.
– The ‘longitudinal Cox model’ utilizes all available observations, resulting in *time‐varying covariates*. Each covariate is assumed to be constant until a new measurement takes place, and the ratio of the hazard function between individuals is a stepwise‐constant function of time. Time‐varying covariates are implemented through the counting‐process data format using the survival package in R [16, 17].

The hazard ratio (HR) for each covariate, with its 95% confidence interval, was obtained by fitting univariate Cox models within each imputed dataset and pooling the resulting estimates using Rubin's rules under both modeling assumptions. Covariates with an HR significantly different from 1 (*p*‐value < 0.05) were classified into risk (HR > 1) or protective (HR < 1) factors. Following this initial classification, *p*‐values were adjusted for multiple testing using the Benjamini–Hochberg false discovery rate procedure.

For risk factors of interest, Kaplan–Meier (K‐M) survival curves were used to display survival, and the log‐rank test was used to compare survival times in each group. Additionally, the violation of the proportional hazards assumption was tested via a Grambsch–Therneau test [18]. In case of a suspected violation, a restricted mean survival time (RMST)‐based test was also utilized to compare survival curves [19, 20]. The RMST represents the expected survival time up to a time point τ, calculated as the area under the K‐M survival curve up to τ. For the choice of τ, we employed both a “fit‐for‐all” value defined as 90% of the minimum of the largest observed follow‐up times (event or censoring) in each group [20], and the time of the last observation in the clinical trial [21].

### 2.6 Variable Selection

To extract jointly informative sets of covariates, we employed elastic net to fit regularized Cox regression models [22].

The method utilizes a mixture of L1‐ and L2‐regularization, which is controlled by the parameter α. We utilized the value α = 0.9 (90% L1‐penalty), allowing the shrinkage of the regression coefficient of uninformative covariates to 0 at appropriate values of the regularization parameter λ. We trained the elastic net model on a long‐format dataset obtained by stacking the multiply imputed datasets, with each observation assigned a weight of 1/50 [23]. Furthermore, we implemented 5‐fold cross‐validation (CV) in which, for each fold, a new dataset was generated using only the training subset to fit the imputation model. Harrell's concordance index (C) [24] was computed in each validation fold for a grid of λ values, averaged, and the largest λ whose mean C lay within one standard error of the maximum mean C was selected (referred to as ‘lambda.1se’ in the glmnet documentation [22]). The C‐index is defined as the proportion of concordant patient pairs among all possible pairs, where a pair is classified as concordant if the patient with the shorter observed survival time is assigned a higher hazard by the model.

We performed 50 iterations of 5‐fold CV to reduce instability in λ selection. Means and corresponding standard errors computed over the iterations are shown in the CV plots, while 2.5^th^–97.5^th^ percentile ranges are presented in the main text as confidence intervals (95% CI). All variables have been scaled to unit variance to ensure fair penalization.

The selected multivariate models were subsequently refitted using standard Cox regression to evaluate the statistical significance of each covariate's regression coefficient.

#### 2.6.1 Time‐Dependent C‐Index

In the longitudinal Cox model, we evaluate the models on the time‐dependent C‐index (C^td^‐index) proposed by Antolini et al. [25], which employs a generalized definition of concordance: a pair of patients i,j, with covariates $X_{i},X_{j}$, and observed survival times $T_{i},T_{j}$, $T_{i}<T_{j}$, are classified as concordant if $\hat{S}\left(T_{i}|X_{i}\right)<\hat{S}\left(T_{i}|X_{j}\right)$, where $\hat{S}\left(t|X_{k}\right)$ is the estimated survival function for patient k according to the Cox model.

### 2.7 Software

All analyses were conducted using R v4.4.2. The ggplot2 (v3.5.0) [26] and survminer (v0.4.9) [27] packages were utilized to generate all plots in this study. Multiple imputation was performed via the mice package (method ‘2l.pmm’) [14]. Cox regression analyses, log‐rank tests, and Grambsch–Therneau tests were performed using the survival package v3.8–3 [17]. RMST testing and plotting were carried out via the survRM2 package v1.0–4 [28]. Regularized Cox models were fitted via the glmnet package v4.1–8 [22].

## 3 Results

### 3.1 Descriptive Statistics

**FIGURE 1:**
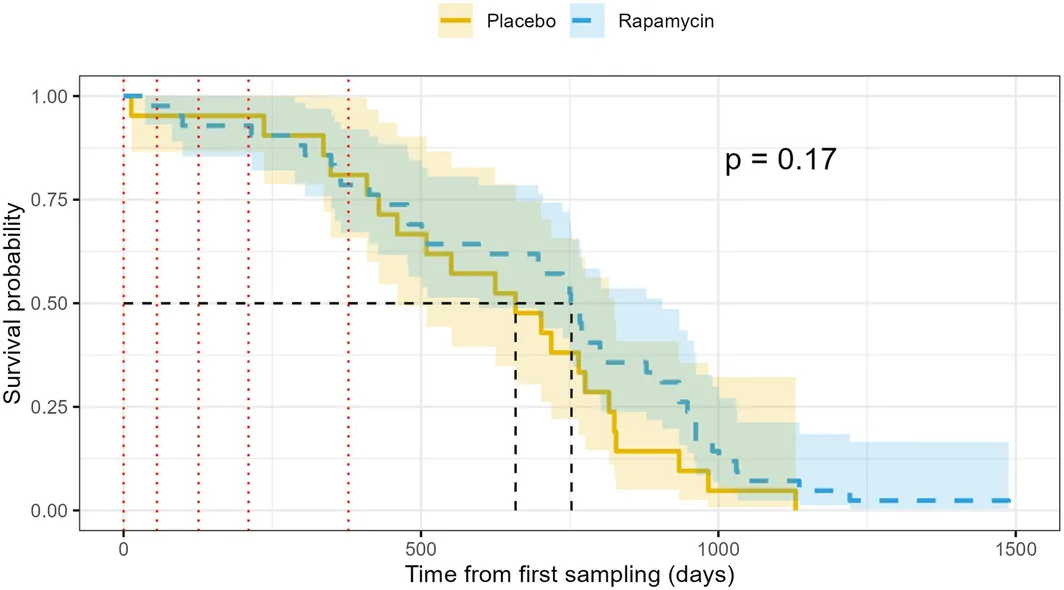
Kaplan–Meier survival estimates for the study cohort, stratified by treatment group, with time zero representing the beginning of the clinical trial. No significant difference in survival curves was observed between placebo and treatment arms (log‐rank *p*‐value 0.17). Vertical red lines indicate sampling times.

Patients presented a median age at screening of 55.4 years (IQR: [44.9, 66.7]) and a median time from onset of 13 months (IQR: [9–16]). Figure 1 shows the Kaplan–Meier estimate of survival probability in the treatment and placebo arms. Median tracheostomy‐free survival from the first sampling was 753 days (IQR: [440, 944]) in the treatment arms, and 659 days in the placebo arm (IQR: [429, 816]). Meanwhile, the median survival time from symptom onset was 1113 days (IQR: [848, 1338]) in the treatment arms and 958 days in the placebo arm (IQR: [855, 1099]). No statistically significant treatment effect on survival was observed in the full cohort (log‐rank *p*‐value = 0.17). Each patient had a recorded survival time, translating to no censoring.

The study population comprised 63 patients (31 males, 49%), with balanced randomization across treatment arms: 13 males received placebo versus 18 in the active treatment group. Bulbar onset was observed in 22% of participants (14/63), equally distributed between placebo (*n* = 6) and treatment arms (*n* = 8). C9orf72 expansions were identified in 11% of cases (7/63), which were predominantly allocated to the treatment arm (86%, 6/7) and exhibited a 43% prevalence of bulbar onset (3/6 treated C9orf72+ patients).

### 3.2 Univariate Cox Regression

**FIGURE 2:**
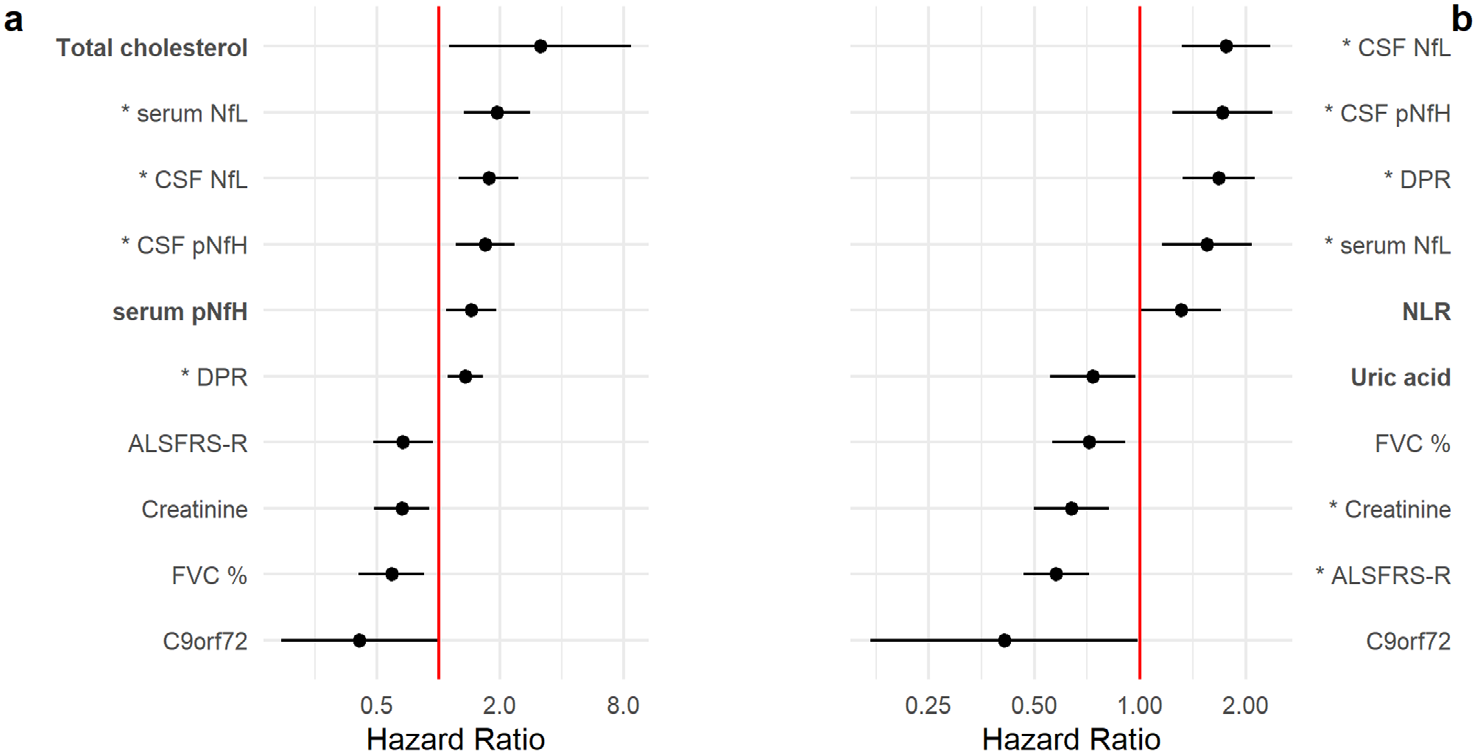
Hazard ratios (HR) of the covariates with a non‐corrected *p*‐value < 0.05 in univariate Cox regression, considering both (a) time‐fixed and (b) time‐varying covariate values. Variables that reached significance in only one Cox model framework are highlighted in bold. Variables marked with a “*” indicate that statistical significance is retained after Benjamini–Hochberg *p*‐value correction. The covariates are listed in order of decreasing HR values. Error bars represent 95% confidence intervals for the HRs. The x‐axis is displayed on a log_2_ scale. Prior to model fitting, all continuous variables have been standardized to z‐scores. Note that categorical variables, being time‐invariant, yield the same estimated HR in both cases; the inclusion of C9orf72 in the right plot is for comparison purposes (NfL: Neurofilament light chain; pNfH: Phosphorylated neurofilament heavy chain; CSF: Cerebrospinal fluid; DPR: Disease progression rate; ALSFRS‐R: ALS functional rating scale revised; NLR: Neutrophil lymphocyte ratio; FVC: Forced vital capacity).

Figure 2 shows the variables with a *p*‐value less than 0.05 in univariate Cox regression, ranked by their HRs. Covariates that remained statistically significant after Benjamini–Hochberg adjustment of *p*‐values are marked with an asterisk (*).

When utilizing time‐fixed covariates (Figure 2a), all neurofilament measures had a significant negative correlation with survival, with 3 out of 4 measures having a higher significance level than the disease progression rate (DPR, measured as the monthly decrease of ALSFRS‐R). Selected risk factors (HR > 1) also included total cholesterol levels. Among the protective factors (HR < 1), we identified FVC and ALSFRS‐R for the clinical scores, the presence of a C9orf72 HRE, and the biomarker creatinine.

Figure 2b displays the significant variables in univariate time‐dependent Cox regression and their associated HRs. Compared with the baseline analysis, the risk factor NLR reached statistical significance, together with the protective variable uric acid.

The analysis identified the C9orf72 expansion as a factor leading to longer survival. Of the seven patients in our cohort with a C9orf72 mutation, six were assigned to treatment arms.

### 3.3 Cox Elastic Net

To extract informative multivariate Cox proportional hazards models under our high‐dimensional setting, we employed elastic net regularization implemented in the R package glmnet. We performed 50 runs of 5‐fold CV to avoid unstable parameter estimates (Figure S2).

**FIGURE 3:**
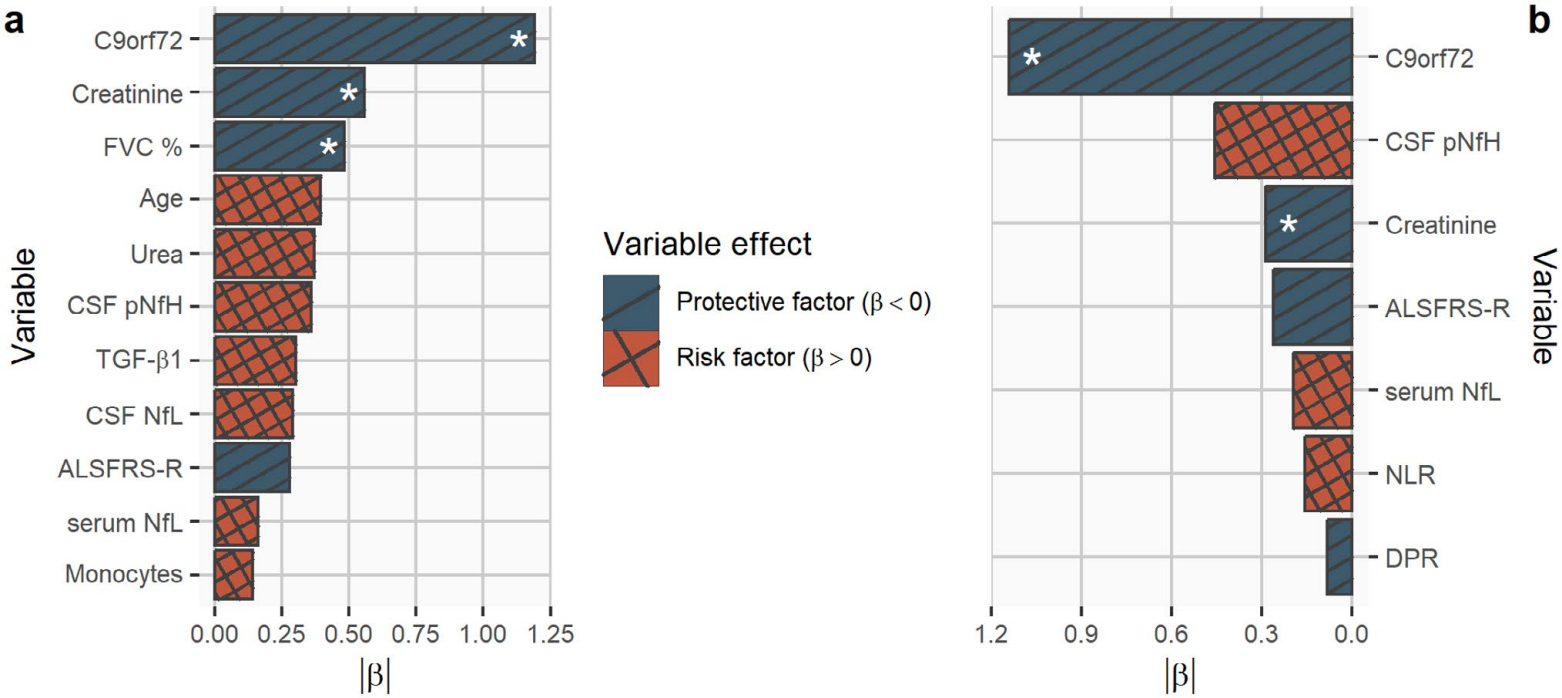
Selected multivariate Cox models via elastic net based on (a) baseline measurements collected at time 0 (baseline Cox model; mean CV C‐index = 0.68; whole‐dataset C‐index = 0.79) and (b) all available repeated measurements (longitudinal Cox model; mean CV C^td^‐index = 0.68; whole‐dataset C^td^‐index = 0.75). Each bar indicates the magnitude of the regression coefficient, color‐coded to indicate whether the covariate is a risk (β > 0, orange) or protective (β < 0, blue) factor. Covariates associated with a significant effect (*p* < 0.05) in the multivariate model are marked by the “*” symbol (NfL: Neurofilament light chain; pNfH: Phosphorylated neurofilament heavy chain; CSF: Cerebrospinal fluid; DPR: Disease progression rate; ALSFRS‐R: ALS functional rating scale revised; NLR: Neutrophil lymphocyte ratio; FVC: Forced vital capacity).

When using the baseline model, elastic net achieved a maximum mean C‐index of 0.68 (95% CI: [0.63, 0.73]) in 5‐fold CV (Figure S2a). Refitting the entire dataset with the optimal regularization parameter (λ) resulted in a multivariate model with 11 predictors (Figure 3a). C9orf72 emerged as a statistically significant covariate (*p* < 0.05), indicating its role as an independent protective factor that is not explained by other measured variables.

Additionally, FVC and creatinine display a significant effect in the multivariate model. Markers of immune activation, namely TGF‐β1 and monocytes, together with the variables age and urea, have been selected as risk factors in the multivariate model despite their non‐significance in the univariate case.

When employing time‐varying covariates, elastic net reached a maximum mean C^td^‐index of 0.68 (95% CI: [0.64, 0.73]) in 5‐fold CV (Figure S2b). The optimal λ selected 7 predictors (Figure 3b), including C9orf72, neurofilament, and creatinine, confirming the results from the baseline case. Among these variables, C9orf72 and creatinine showed significance in the multivariate context. Moreover, NLR and DPR were selected only in this time‐dependent setting.

### 3.4 Survival Time Analysis of C9orf72+ Within RAP‐ALS Data

**FIGURE 4:**
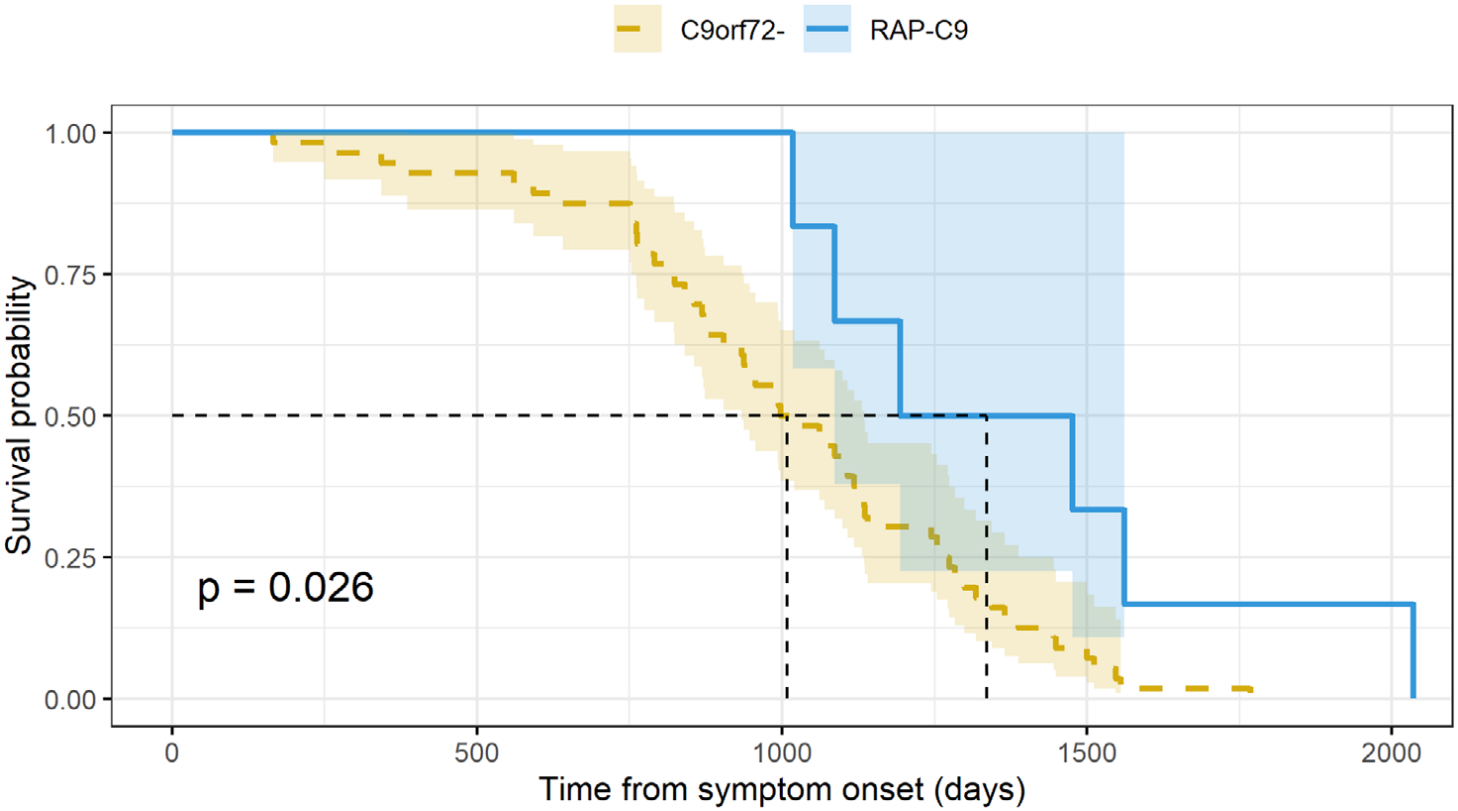
Kaplan–Meier estimates of survival function within the RAP‐ALS cohort. The blue line indicates treated C9orf72+ patients (*n* = 6); the yellow line indicates C9orf72‐ patients (*n* = 56). The *p*‐value refers to the log‐rank test between the two strata.

The observed protective effect in treated C9orf72+ patients (RAP‐C9) prompted further investigation within this subgroup. Comparison of RAP‐C9 (*n* = 6) and C9ORF72‐ (*n* = 56) patients within our cohort confirmed significantly improved survival outcomes (log‐rank *p* = 0.026), as demonstrated in the Kaplan–Meier analysis (Figure 4).

### 3.5 Comparison of C9orf72+ Survival With Independent Data

To evaluate rapamycin's therapeutic potential in the C9orf72+ population, we compared outcomes from the 6 RAP‐C9 patients with an independent C9orf72+ control cohort (CTRL‐C9). To minimize selection bias arising from differing data collection protocols, only CTRL‐C9 patients who were event‐free and still under follow‐up after 15 months (corresponding to the median time to first sampling among the RAP‐C9 patients) were included. Moreover, patients exceeding a 19‐month diagnostic delay were excluded to align with the trial's inclusion criteria.

After filtering for bias, the control dataset included 40 patients, with 8 censored survival times, and a median survival time from onset (Kaplan–Meier estimate) of 1029 days.

No significant difference was observed between the survival curves of RAP‐C9 and CTRL‐C9 using the log‐rank test (Figure S3).

Next, we evaluated whether the proportional hazards assumption was met when comparing the two groups. Although the Grambsch–Therneau test [18] did not indicate a significant violation (*p* = 0.06), the plot of scaled Schoenfeld residuals (Figure S4) suggested a time‐dependent pattern in the Cox regression coefficient for the treatment. Specifically, the coefficient showed negative values at earlier times, consistent with the timing of the intervention.

**FIGURE 5:**
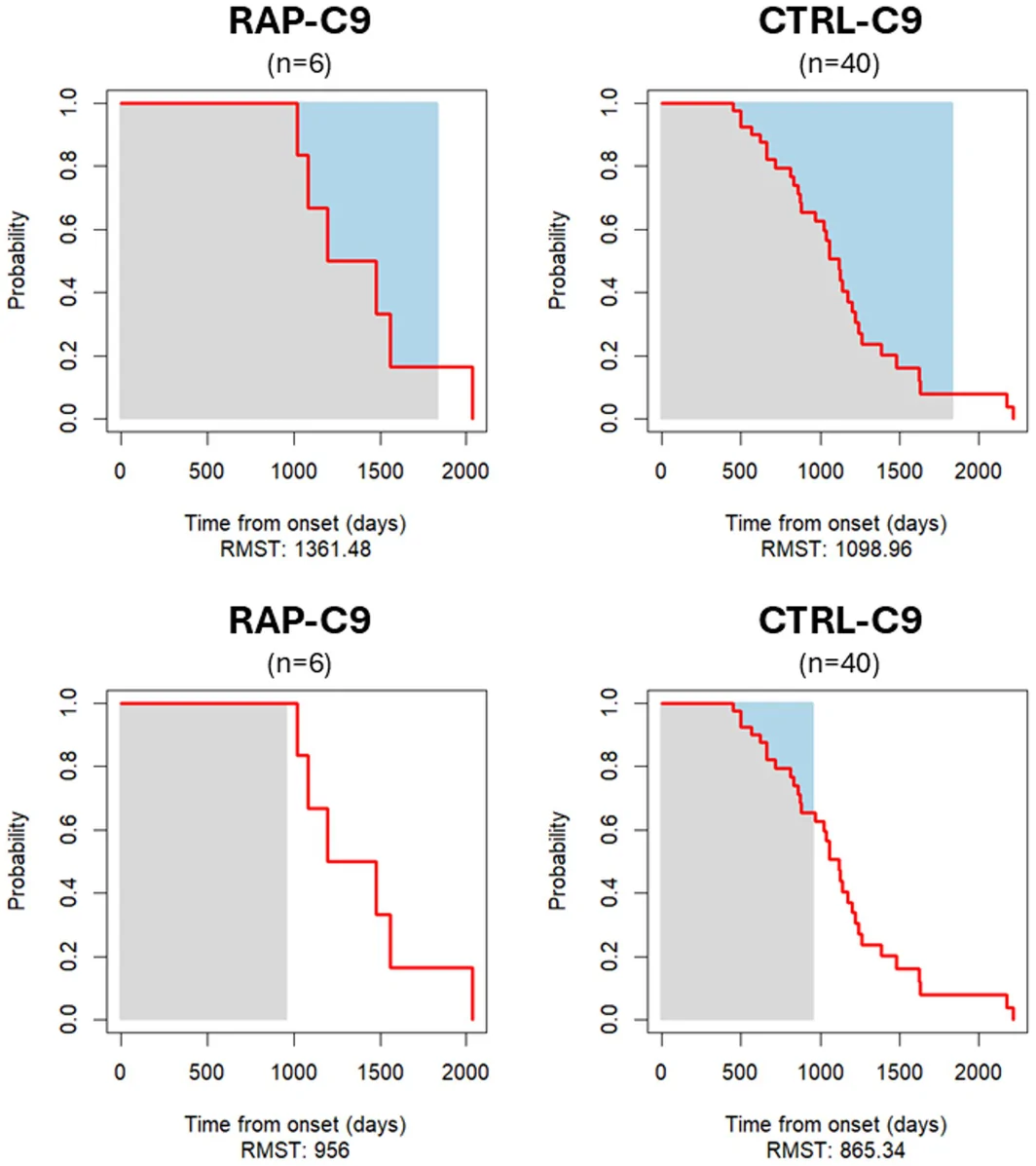
Comparison of RMST between RAP‐C9 (left, *n* = 6) and CTRL‐C9 (right, *n* = 40). Using both τ = 1832 days (top) and τ = 956 days (bottom) demonstrated a significant effect (*p* = 0.039 and *p* < 0.0001, respectively).

To account for a potential violation of the proportional hazards assumption, we compared the RMST between the two cohorts (Figure 5). When comparing the survival curves up to τ = 956 days (the latest follow‐up time among RAP‐C9 patients), we observed a significantly lower RMST in the CTRL‐C9 cohort (*p* < 0.0001). This difference remained significant when using τ = 1832 days (corresponding to 90% of the minimum of the latest observed event times in the two groups; *p* = 0.039), even if it corresponds to a later time than the end of the study and the last rapamycin administration. Notably, however, the RAP‐C9 cohort had a higher mean baseline ALSFRS‐R score than both the CTRL‐C9 cohort (41.0 vs. 38.8; *p* = 0.16) and the remaining RAP‐ALS participants (41.0 vs. 38.4; *p* = 0.04; Figure S5).

## 4 Discussion

We performed a comprehensive survival analysis using data from a clinical trial of rapamycin. Through univariate analysis and multivariate Cox regression modeling, we assessed the impact of clinical and biological covariates, treating them as both fixed and time‐varying factors. Several covariates emerged as significant predictors of tracheostomy‐free survival in the univariate analysis (corrected *p*‐value < 0.05), including neurofilament levels, creatinine, DPR, and ALSFRS‐R score. These findings align with previously reported prognostic markers in ALS [29, 30, 31, 32, 33, 34, 35, 36].

Both neurofilament species—specifically, NfL in both serum and CSF and pNfH in CSF—demonstrated statistical significance in univariate analyses (corrected *p*‐value < 0.05; HR > 1). Notably, both multivariate models (Figure 3) included serum NfL and CSF pNfH, suggesting that neurofilament light and heavy chains provide complementary, rather than fully overlapping, information. The inclusion of serum neurofilament in the longitudinal model, despite CSF neurofilament being a more direct indicator of axonal damage, may be due to its association with blood volume, which is correlated with lean body mass [37, 38], thus offering indirect information about the degree of muscular atrophy.

Moreover, we found that the neutrophil‐lymphocyte ratio, a marker of acute inflammation [39], was selected only when its entire time‐dependent trajectory was considered, suggesting it as a potential late‐stage risk factor; however, because its corrected *p*‐value was non‐significant, its association with survival should be investigated in a larger cohort.

Elastic net regression demonstrated moderate generalizability across both baseline (mean CV C‐index: 0.68) and longitudinal (mean CV C^td^‐index: 0.68) modeling frameworks. Both selected multivariate models included either the ALSFRS‐R score or DPR, highlighting the importance of this functional scale as a prognostic factor. At the same time, both models incorporated biomarkers representing three key ALS hallmarks: Neurofilaments (NfL and pNfH) as markers of axonal degeneration, creatinine reflecting muscle mass, and proinflammatory immune activation markers. Nonetheless, these findings should be interpreted cautiously. The models lack independent external validation, and their generalizability is limited by the small RAP‐ALS sample, specific eligibility criteria, and risk of overfitting. Confidence in the findings is further limited by the extent of missing data; CSF biomarkers, which were collected at only two protocol‐defined time points, should be interpreted as approximately time‐invariant rather than as fully longitudinal covariates. Therefore, given the post hoc and hypothesis‐generating nature of this study, the selected biomarkers are not suitable for direct clinical application. Instead, these results may guide future survival studies by suggesting that the integration of measures reflecting axonal damage, neuroinflammation, and muscle degeneration may provide greater prognostic discrimination for tracheostomy‐free survival than considering individual biological domains in isolation.

Our analysis revealed a protective association of the *C9orf72* HRE in both univariate and multivariate models, despite it being associated with a more aggressive phenotype in the literature [40, 41, 42]. Notably, 6 out of 7 *C9orf72*+ patients received rapamycin, suggesting a potential genotype‐specific treatment effect, as supported by log‐rank testing within the RAP‐ALS cohort (*p* = 0.026). Although the comparison with the CTRL‐C9 cohort (*n* = 40) did not reach significance using the log‐rank test, we observed a protective effect of rapamycin in the restricted mean survival time (RMST) analysis (*p* = 0.039 and *p* < 0.0001 with τ = 1832 and τ = 956 days, respectively). RMST‐based testing is recognized for its increased statistical power in the presence of non‐proportional hazards, which is consistent with our study context, as rapamycin's 18‐week administration period suggests that any protective effect may be limited to the early disease stages.

Mechanistically, these observations align with emerging evidence linking *C9orf72* to autophagy regulation through mTOR‐mediated pathways [43]. The literature suggests that a C9orf72 HRE can cause ALS through both toxic dipeptide repeat proteins and autophagy impairment due to C9orf72 loss‐of‐function [44, 45, 46, 47], and boosting protein clearance via mTOR inhibition could potentially address both processes. Rapamycin has already shown beneficial effects in preclinical models of both C9orf72 loss‐of‐function and toxicity [46, 47, 48]. However, the interaction between the mTOR pathway and C9orf72 remains debated [44], and any mechanistic hypothesis requires further validation through additional studies.

Additionally, the RAP‐C9 findings warrant cautious interpretation. This subgroup analysis was based on only six treated C9orf72 mutation carriers, conducted post hoc across multiple exploratory analyses, and showed imbalance in treatment allocation; therefore, the observed association may reflect random variation or the effect of confounding factors. Moreover, treated C9orf72 carriers had a higher mean baseline ALSFRS‐R score than both CTRL‐C9 (41.0 vs. 38.8; *p* = 0.16) and the remaining RAP‐ALS cohort (41.0 vs. 38.4; *p* = 0.04; Figure S5), suggesting more favorable functional status at enrolment. Differences in selection criteria, follow‐up structure, and censoring patterns between the RAP‐ALS and CTRL‐C9 cohorts further limit the validity of between‐cohort comparisons. Consequently, these observations should be regarded as hypothesis‐generating only, and confirmation would require adequately powered prospective studies.

Despite these limitations, the present study highlights the value of post hoc analyses of longitudinal data from an overall negative clinical trial. Integrating clinical trajectories with molecular and genetic measures can identify patient subgroups and generate biologically plausible hypotheses. Accordingly, the present work does not support immediate clinical implementation but may inform the design of future ALS trials, while offering a methodological framework for future post hoc analyses.

## Author Contributions

**Alex De Nardi:** investigation, writing – original draft, methodology, validation, visualization, writing – review and editing, software, formal analysis, conceptualization, data curation. **Alessio Paris:** conceptualization, investigation, writing – review and editing, supervision, validation. **Mario Lauria:** conceptualization, writing – review and editing, supervision, validation. **Ilaria Martinelli:** writing – review and editing, data curation, validation. **Elisabetta Zucchi:** writing – review and editing, data curation, validation. **Cecilia Simonini:** writing – review and editing, data curation, validation. **Jessica Mandrioli:** conceptualization, funding acquisition, writing – review and editing, supervision, data curation, resources, validation. **Luca Marchetti:** conceptualization, investigation, funding acquisition, writing – review and editing, project administration, validation, supervision, resources.

## Funding

The RAP‐ALS study was supported by ARISLA (Fondazione Italiana di Ricerca per la SLA) (FGCR02/2015 to J.M.) and Pfizer Inc. (drug furniture) (grant no. 53232941, program title: to J.M.). The Emilia Romagna Registry for ALS (ERRALS) is supported by a grant from the Emilia Romagna Regional Health Authority (GPG/2022/1343).

## Conflicts of Interest

J.M. reports the following: consulting for Biogen, Amylyx, Zambon, Maat Pharma, Italfarmaco; research support from Pfizer, Biogen, Ionis, Roche, Apellis, Ferrer, ABScience, Amylix, PTC Therapeutics, Novartis, Prilenia, Dianthus Therapeutics. A.D.N., A.P., M.L., I.M., E.Z., C.S., and L.M. declare that they have no Conflicts of Interest.

## Supporting information


**Table S1:** List of covariates utilized in the analysis. The percentage of missing measurements (NA), calculated across all patient visits, is also presented.
**Table S2:** RAP‐ALS study group.
**Table S3:** Members of the ERRALS Group.
**Figure S1:** Distribution of between‐patient and within‐patient variability in CSF measures, quantified as (i) the deviation between each patient's mean value and the overall cohort mean, and (ii) the deviation between longitudinal measurements and their corresponding patient‐specific mean (for patients with two observations), respectively.
**Figure S2:** Repeated 5‐fold CV (blue) and training (black) performance of the glmnet‐fitted multivariate Cox models at different values of the regularization parameter λ. For the baseline Cox model (left), the selected model comprises 11 covariates, associated with a CV C‐index of 0.68 (whole‐dataset C‐index = 0.79). For the longitudinal Cox model (right), the time‐dependent version of the C‐index was employed, achieving a mean CV value of 0.68 and leading to the selection of a multivariate model including 7 predictors (whole‐dataset Ctd‐index = 0.75). The plots show the mean values and standard error ranges (shaded area) over 50 iterations. The number of variables included in the multivariate model at different values of the regularization parameter λ is also shown.
**Figure S3:** Kaplan–Meier survival estimates of RAP‐C9 versus CTRL‐C9, after filtering to correct sampling‐related biases. No significant difference between the two groups has been detected via the log‐rank test (p = 0.44).
**Figure S4:** Plot of scaled Schoenfeld residuals. The estimated Cox regression coefficient versus time (solid line) suggests time‐dependent dynamics that are coherent with treatment administration timings (p = 0.06).
**Figure S5:** Comparison of baseline ALSFRS‐R score distribution within cohorts. The RAP‐C9 cohort showed a higher mean baseline ALSFRS‐R score (41.0, *n* = 6) than both the non‐RAP‐C9 participants within RAP‐ALS (38.4, *n* = 57; Welch's t‐test p = 0.048) and the CTRL‐C9 cohort (38.8, *n* = 25; p = 0.156). Only 25 out of 40 CTRL‐C9 patients are considered due to substantial missingness in ALSFRS‐R measures. Black dots indicate mean values.

## Data Availability

The data that support the findings of this study are available to external researchers who provide methodologically sound scientific proposals and whose proposed use of the data has been approved by an independent review committee identified for this purpose. A materials transfer and/or data access agreement with the study promoter will be required for accessing shared data. However, individual participant data will not be available because informed consent did not explicitly include this.

## References

[1] K. C. Arthur , A. Calvo , T. R. Price , J. T. Geiger , A. Chiò , and B. J. Traynor , “Projected Increase in Amyotrophic Lateral Sclerosis From 2015 to 2040,” Nature Communications 7, no. 1 (2016): 1–6. 10.1038/ncomms12408 PMC4987527 27510634

[2] T. M. Miller , M. E. Cudkowicz , A. Genge , et al., “Trial of Antisense Oligonucleotide Tofersen for SOD1 ALS,” New England Journal of Medicine 387, no. 12 (2022): 1099–1110, 10.1056/NEJMOA2204705. 36129998

[3] J. Mandrioli , S. A. Malerba , E. Beghi , et al., “Riluzole and Other Prognostic Factors in ALS: A Population‐Based Registry Study in Italy,” Journal of Neurology 265, no. 4 (2018): 817–827, 10.1007/S00415-018-8778-Y. 29404735

[4] J. Mandrioli , R. D'Amico , E. Zucchi , et al., “Randomized, Double‐Blind, Placebo‐Controlled Trial of Rapamycin in Amyotrophic Lateral Sclerosis,” Nature Communications 14, no. 1 (2023): 4970, 10.1038/s41467-023-40734-8. PMC10435464 37591957

[5] A. Chiò , G. Logroscino , O. Hardiman , et al., “Prognostic Factors in ALS: A Critical Review,” Amyotrophic Lateral Sclerosis: Official Publication of the World Federation of Neurology Research Group on Motor Neuron Diseases 10, no. 5–6 (2009): 310–323, 10.3109/17482960802566824. PMC3515205 19922118

[6] A. Calvo , C. Moglia , C. Lunetta , et al., “Factors Predicting Survival in ALS: A Multicenter Italian Study,” Journal of Neurology 264, no. 1 (2017): 54–63, 10.1007/S00415-016-8313-Y. 27778156

[7] C. N. Fournier , V. James , and J. D. Glass , “Clinically Meaningful Change: Evaluation of the Rasch‐Built Overall Amyotrophic Lateral Sclerosis Disability Scale (ROADS) and the ALSFRS‐R,” Amyotrophic Lateral Sclerosis and Frontotemporal Degeneration 24, no. 3–4 (2023): 311–316, 10.1080/21678421.2022.2153607. 36476010

[8] D. Krawczuk , A. Kulczyńska‐Przybik , and B. Mroczko , “Clinical Application of Blood Biomarkers in Neurodegenerative Diseases—Present and Future Perspectives,” International Journal of Molecular Sciences 25, no. 15 (2024): 8132, 10.3390/IJMS25158132. PMC11311320 39125699

[9] Y. M. Falzone , T. Domi , A. Mandelli , et al., “Integrated Evaluation of a Panel of Neurochemical Biomarkers to Optimize Diagnosis and Prognosis in Amyotrophic Lateral Sclerosis,” European Journal of Neurology 29, no. 7 (2022): 1930–1939, 10.1111/ENE.15321. PMC9314044 35263489

[10] F. Faghri , F. Brunn , A. Dadu , et al., “Identifying and Predicting Amyotrophic Lateral Sclerosis Clinical Subgroups: A Population‐Based Machine‐Learning Study,” Lancet Digital Health 4, no. 5 (2022): e359–e369, 10.1016/S2589-7500(21)00274-0. PMC9038712 35341712

[11] L. H. Kuan , P. Parnianpour , R. Kushol , et al., “Accurate Personalized Survival Prediction for Amyotrophic Lateral Sclerosis Patients,” Scientific Reports 13, no. 1 (2023): 1–7, 10.1038/s41598-023-47935-7. PMC10673879 38001260

[12] J. Mandrioli , E. Zucchi , I. Martinelli , et al., “Factors Predicting Disease Progression in C9ORF72 ALS Patients,” Journal of Neurology 270, no. 2 (2023): 877–890, 10.1007/S00415-022-11426-Y. 36280624

[13] A. Ghezzi , G. Gianferrari , E. Baldassarri , et al., “Phenotypical Characterization of C9ALS Patients From the Emilia Romagna Registry of ALS: A Retrospective Case–Control Study,” Genes 16, no. 3 (2025): 309, 10.3390/GENES16030309/S1. PMC11942173 40149460

[14] S. van Buuren and K. Groothuis‐Oudshoorn , “Mice: Multivariate Imputation by Chained Equations in R,” Journal of Statistical Software 45, no. 3 (2011): 1–67.

[15] D. R. Cox , “Regression Models and Life‐Tables,” Journal of the Royal Statistical Society, Series B: Statistical Methodology 34, no. 2 (1972): 187–202, 10.1111/J.2517-6161.1972.TB00899.X.

[16] Z. Zhang , J. Reinikainen , K. A. Adeleke , M. E. Pieterse , and C. G. M. Groothuis‐Oudshoorn , “Time‐Varying Covariates and Coefficients in Cox Regression Models,” Annals of Translational Medicine 6, no. 7 (2018): 121, 10.21037/ATM.2018.02.12. PMC6015946 29955581

[17] T. Therneau , A Package for Survival Analysis in R. R package version 3.8–3, 2024 accessed at, https://CRAN.R‐project.org/package=survival.

[18] P. M. Grambsch and T. M. Therneau , “Proportional Hazards Tests and Diagnostics Based on Weighted Residuals,” Biometrika 81, no. 3 (1994): 515–526, 10.1093/BIOMET/81.3.515.

[19] H. Uno , B. Claggett , L. Tian , et al., “Moving Beyond the Hazard Ratio in Quantifying the Between‐Group Difference in Survival Analysis,” Journal of Clinical Oncology 32, no. 22 (2014): 2380–2385, 10.1200/JCO.2014.55.2208. PMC4105489 24982461

[20] P. Royston and M. K. B. Parmar , “The Use of Restricted Mean Survival Time to Estimate the Treatment Effect in Randomized Clinical Trials When the Proportional Hazards Assumption Is in Doubt,” Statistics in Medicine 30, no. 19 (2011): 2409–2421, 10.1002/SIM.4274. 21611958

[21] L. Tian , H. Jin , H. Uno , et al., “On the Empirical Choice of the Time Window for Restricted Mean Survival Time,” Biometrics 76, no. 4 (2020): 1157–1166, 10.1111/BIOM.13237. PMC8687138 32061098

[22] N. Simon , J. Friedman , T. Hastie , and R. Tibshirani , “Regularization Paths for Cox's Proportional Hazards Model via Coordinate Descent,” Journal of Statistical Software 39, no. 5 (2011): 1–13, 10.18637/jss.v039.i05. PMC4824408 27065756

[23] Y. Wan , S. Datta , D. J. Conklin , and M. Kong , “Variable Selection Models Based on Multiple Imputation With an Application for Predicting Median Effective Dose and Maximum Effect,” Journal of Statistical Computation and Simulation 85, no. 9 (2015): 1902–1916, 10.1080/00949655.2014.907801. PMC4583148 26412909

[24] F. E. Harrell , R. M. Califf , D. B. Pryor , K. L. Lee , and R. A. Rosati , “Evaluating the Yield of Medical Tests,” Journal of the American Medical Association 247, no. 18 (1982): 2543–2546, 10.1001/JAMA.1982.03320430047030. 7069920

[25] L. Antolini , P. Boracchi , and E. Biganzoli , “A time‐dependent discrimination index for survival data,” Statistics in Medicine 24, no. 24 (2005): 3927–3944, 10.1002/SIM.2427. 16320281

[26] H. Wickham , ggplot2: Elegant Graphics for Data Analysis (Springer‐Verlag New York, 2016).

[27] A. Kassambara , M. Kosinski , and P. Biecek , survminer: Drawing Survival Curves using 'ggplot2'. R package version 0.5.0, 2024.

[28] H. Uno , L. Tian , M. Horiguchi , A. Cronin , C. Battioui , and J. Bell , survRM2: Comparing Restricted Mean Survival Time. R package version 1.0–4, 2022 accessed at, https://CRAN.R‐project.org/package=survRM2.

[29] N. Hertel , M. Kuzma‐Kozakiewicz , M. Gromicho , et al., “Analysis of Routine Blood Parameters in Patients With Amyotrophic Lateral Sclerosis and Evaluation of a Possible Correlation With Disease Progression—A Multicenter Study,” Frontiers in Neurology 13 (2022): 940375, 10.3389/FNEUR.2022.940375. PMC9364810 35968316

[30] M. R. Janse Van Mantgem , W. van Rheenen , A. v. Hackeng , et al., “Association Between Serum Lipids and Survival in Patients With Amyotrophic Lateral Sclerosis: A Meta‐Analysis and Population‐Based Study,” Neurology 100, no. 10 (2023): E1062–E1071, 10.1212/WNL.0000000000201657. PMC9990853 36460467

[31] F. Zhang , Q. Zhang , Y. Ke , et al., “Serum Uric Acid Levels in Patients With Amyotrophic Lateral Sclerosis: A Meta‐Analysis,” Scientific Reports 8, no. 1 (2018): 1–6, 10.1038/s41598-018-19609-2. PMC5773600 29348425

[32] A. Czaplinski , A. A. Yen , and S. H. Appel , “Forced Vital Capacity (FVC) as an Indicator of Survival and Disease Progression in an ALS Clinic Population,” Journal of Neurology, Neurosurgery, and Psychiatry 77, no. 3 (2006): 390, 10.1136/JNNP.2005.072660. PMC2077717 16484652

[33] F. Kimura , C. Fujimura , S. Ishida , et al., “Progression Rate of ALSFRS‐R at Time of Diagnosis Predicts Survival Time in ALS,” Neurology 66, no. 2 (2006): 265–267, 10.1212/01.WNL.0000194316.91908.8A. 16434671

[34] W. M. Su , Y. F. Cheng , Z. Jiang , et al., “Predictors of Survival in Patients With Amyotrophic Lateral Sclerosis: A Large Meta‐Analysis,” eBioMedicine 74 (2021): 103732, 10.1016/J.EBIOM.2021.103732/. PMC8646173 34864363

[35] F. Verde , S. Licaj , D. Soranna , N. Ticozzi , V. Silani , and A. Zambon , “Cerebrospinal Fluid and Blood Neurofilament Light Chain Levels in Amyotrophic Lateral Sclerosis and Frontotemporal Degeneration: A Meta‐Analysis,” European Journal of Neurology 31, no. 9 (2024): e16371, 10.1111/ENE.16371. PMC11295179 38937912

[36] F. Verde , M. Otto , and V. Silani , “Neurofilament Light Chain as Biomarker for Amyotrophic Lateral Sclerosis and Frontotemporal Dementia,” Frontiers in Neuroscience 15 (2021): 15, 10.3389/FNINS.2021.679199. PMC8255624 34234641

[37] P. Boer , “Estimated Lean Body Mass as an Index for Normalization of Body Fluid Volumes in Humans,” American Journal of Physiology. Renal Physiology 247, no. 4 Pt 2 (1984): F632–F636, 10.1152/AJPRENAL.1984.247.4.F632. 6496691

[38] A. Raes , S. van Aken , M. Craen , R. Donckerwolcke , and J. vande Walle , “A Reference Frame for Blood Volume in Children and Adolescents,” BMC Pediatrics 6 (2006): 3, 10.1186/1471-2431-6-3. PMC1434736 16503982

[39] M. A. Leone , J. Mandrioli , S. Russo , et al., “Neutrophils‐To‐Lymphocyte Ratio Is Associated With Progression and Overall Survival in Amyotrophic Lateral Sclerosis,” Biomedicine 10, no. 2 (2022): 354, 10.3390/BIOMEDICINES10020354/S1. PMC8962424 35203564

[40] H. J. Westeneng , T. P. A. Debray , A. E. Visser , et al., “Prognosis for Patients With Amyotrophic Lateral Sclerosis: Development and Validation of a Personalised Prediction Model,” Lancet Neurology 17, no. 5 (2018): 423–433, 10.1016/S1474-4422(18)30089-9. 29598923

[41] H. Malkki , “ *C9orf72* Repeat Expansion Linked to Aggressive Disease in Male Patients With Spinal‐Onset ALS,” Nature Reviews. Neurology 12 (2016): 616, 10.1038/nrneurol.2016.161. 27739539

[42] F. Trojsi , M. Siciliano , C. Femiano , et al., “Comparative Analysis of C9Orf72 and Sporadic Disease in a Large Multicenter ALS Population: The Effect of Male Sex on Survival of C9Orf72 Positive Patients,” Frontiers in Neuroscience 13, no. 5 (2019): 456655, 10.3389/FNINS.2019.00485. PMC6534038 31156370

[43] R. Diab , F. Pilotto , and S. Saxena , “Autophagy and Neurodegeneration: Unraveling the Role of C9ORF72 in the Regulation of Autophagy and Its Relationship to ALS‐FTD Pathology,” Frontiers in Cellular Neuroscience 17 (2023): 1086895, 10.3389/FNCEL.2023.1086895. PMC10060823 37006471

[44] Z. Z. Chong , D. L. Menkes , and N. Souayah , “Pathogenesis Underlying Hexanucleotide Repeat Expansions in C9orf72 Gene in Amyotrophic Lateral Sclerosis,” Reviews in the Neurosciences 35, no. 1 (2024): 85–97, 10.1515/REVNEURO-2023-0060. 37525497

[45] Q. Zhu , J. Jiang , T. F. Gendron , et al., “Reduced C9ORF72 Function Exacerbates Gain of Toxicity From ALS/FTD‐Causing Repeat Expansion in C9orf72,” Nature Neuroscience 23, no. 5 (2020): 615–624, 10.1038/s41593-020-0619-5. PMC7384305 32284607

[46] M. Pu , Y. Tai , L. Yuan , et al., “The Contribution of Proteasomal Impairment to Autophagy Activation by C9orf72 Poly‐GA Aggregates,” Cellular and Molecular Life Sciences 79, no. 9 (2022): 501, 10.1007/S00018-022-04518-5. PMC11803000 36036324

[47] H. de Calbiac , S. Renault , G. Haouy , et al., “Poly‐GP Accumulation due to C9orf72 Loss of Function Induces Motor Neuron Apoptosis Through Autophagy and Mitophagy Defects,” Autophagy 20, no. 10 (2024): 2164–2185, 10.1080/15548627.2024.2358736. PMC11423671 39316747

[48] Q. Shao , M. Yang , C. Liang , et al., “C9orf72 and smcr8 Mutant Mice Reveal MTORC1 Activation due to Impaired Lysosomal Degradation and Exocytosis,” Autophagy 16, no. 9 (2019): 1635–1650, 10.1080/15548627.2019.1703353. PMC8386604 31847700

