# Supplementary material for: Multivariate Survival Analysis of a Comprehensive Clinical Trial of Rapamycin in Amyotrophic Lateral Sclerosis Explores Prognostic Factors and Survival Patterns in C9orf72 Carriers
Source: Eur J Neurol. 2026 Sep 19;33(9):e70756. doi: 10.1111/ene.70756 (PMC13589253; doi:10.1111/ene.70756)
Supplement: Supplementary file 1 [file ENE-33-e70756-s001.docx]

## **Multivariate survival analysis of a comprehensive clinical trial of rapamycin in amyotrophic lateral sclerosis explores prognostic factors and survival patterns in C9orf72 carriers**

## Alex De Nardi, Alessio Paris, Mario Lauria, Ilaria Martinelli, Elisabetta Zucchi, Cecilia Simonini, Jessica Mandrioli, Luca Marchetti

## **Supplementary File S1**

| **Name** | **Unit** | **%NA** |
| --- | --- | --- |
| serum pNfH | ng/ml | 7 |
| CSF pNfH | ng/ml | 64 |
| serum NfL | ng/ml | 7 |
| CSF NfL | ng/ml | 64 |
| Randomization arm | - | 0 |
| Sex: male | - | 0 |
| BMI | kg/m^2^ | 1 |
| Red blood cells | 10^6^/mmc | 5 |
| Platelets | 10^3^/mmc | 5 |
| Leukocytes | 10^3^/mmc | 5 |
| Neutrophils | 10^3^/mmc | 5 |
| Lymphocytes | 10^3^/mmc | 5 |
| Monocytes | 10^3^/mmc | 5 |
| Basophils | 10^3^/mmc | 5 |
| Eosinophils | 10^3^/mmc | 5 |
| Hemoglobin | g/dl | 5 |
| Hematocrit | % | 5 |
| Creatinine | mg/dl | 5 |
| Albumin | g/dl | 5 |
| Gamma globulin | % | 9 |
| C reactive protein | mg/dl | 6 |
| FVC | % | 9 |
| Time from onset | months | 0 |
| Disease progression rate (DPR) | points/month | 0 |
| Creatine kinase | IU/L | 9 |
| Total cholesterol | mg/dl | 14 |
| LDL | mg/dl | 16 |
| HDL | mg/dl | 16 |
| Triglycerides | mg/dl | 16 |
| Uric acid | mg/dl | 18 |
| Urea | mg/dl | 8 |

| **Name** | **Unit** | **%NA** |
| --- | --- | --- |
| Ferritin | ng/ml | 10 |
| Vitamin D | ng/ml | 15 |
| TGF-β1 | pg/ml | 11 |
| IL-12 | pg/ml | 37 |
| IL-18 | pg/ml | 11 |
| TNF-α | pg/ml | 43 |
| CD3+ | CD3 | 12 |
| CD4+ | CD4+ | 12 |
| CD4+ CD38+ DR+ | u/l | 39 |
| CD4 CXCR3+ | u/l | 42 |
| CD4+ CD39+ | u/l | 34 |
| CD4+ PD1+ | u/l | 34 |
| T-reg CD4+ | u/l | 12 |
| T-reg CD4+ CD38+ DR+ | u/l | 39 |
| T-reg CD4+ CXCR3+ | u/l | 39 |
| T-reg CD4+ CD39+ | u/l | 35 |
| T-reg CD4+ PD1+ | u/l | 35 |
| CD8+ | u/l | 36 |
| CD8+ CD38+ DR+ | u/l | 41 |
| CD8+ CXCR3+ | u/l | 41 |
| CD8+ CD39+ | u/l | 36 |
| CD8+ PD1+ | u/l | 36 |
| T-reg CD8+ | u/l | 36 |
| T-reg CD8+ CD38+ DR+ | u/l | 40 |
| T-reg CD8+ CXCR3+ | u/l | 40 |
| T-reg CD8+ CD39+ | u/l | 36 |
| T-reg CD8+ PD1+ | u/l | 38 |
| Age | years | 0 |
| ALSFRS-R | points | 0 |
| NLR | ratio | 5 |
| C9orf72 status | - | 0 |

**Table S1** List of covariates utilized in the analysis. The percentage of missing measurements (NA), calculated across all patient visits, is also presented.

| **RAP-ALS sites and locations** | **Investigators** |
| --- | --- |
| ALS Center, Azienda Ospedaliero Universitaria di Modena, Modena, Italy | Jessica Mandrioli  Elisabetta Zucchi  Ilaria Martinelli  Cecilia Simonini  Giulia Gianferrari  Nicola Fini  Roberta Bedin  Annalisa Gessani  Francesca Prompicai  Silvia Parisi  Daniela Gallesi  Andrea Ghezzi |
| Statistical Unit of Modena, University of Modena and Reggio Emilia, Italy | Roberto D’Amico  Federico Banchelli  Riccardo Cuoghi Costantini |
| Immunology Lab, University of Modena and Reggio Emilia, Italy | Andrea Cossarizza  Sara De Biasi  Domenico Lo Tartaro  Anita Neroni  Marcello Pinti |
| ALS Center, Maggiore della Carità Hospital, University of Piemonte Orientale, Novara, Italy | Letizia Mazzini  Ada Scognamiglio  Fabiola De Marchi  Paola Odoli |
| Centro Regionale Esperto per la SLA (CRESLA), Azienda Ospedaliero Universitaria Città della Salute e della Scienza, University of Turin, Turin, Italy | Adriano Chiò  Andrea Calvo  Cristina Moglia |
| IRCCS Istituto Neurologico Carlo Besta di Milano, Milan, Italy | Giuseppe Lauria Pinter  Eleonora Dalla Bella  Raffaella Lombardi  Enrica Bersano |
| NEuroMuscular Omnicentre (NEMO), Fondazione Serena Onlus, Milan, Italy | Christian Lunetta  Claudia Tarlarini  Francesca Gerardi  Valeria Sansone |
| Neurological Clinic, IRCCS Ospedale Policlinico San Martino, Genoa, Italy | Claudia Caponnetto  Giuseppe Meo  Corrado Cabona  Chiara Gemelli |
| Centro per la diagnosi, cura e ricerca nella SLA e Malattie del Motoneurone, Azienda Ospedale Università di Padova, Padua, Italy | Gianni Sorarù  Andrea Fortuna |

***Table S2*** *RAP-ALS study group.*

**ERRALS Group**

**Project coordinator:** Prof. J. Mandrioli

**Collaborating centers:**

- Department of Neurosciences, Azienda Ospedaliero Universitaria di Modena and Department of Biomedical, Metabolic and Neural Sciences, University of Modena and Reggio Emilia, Modena, Italy (Jessica Mandrioli, Nicola Fini, Ilaria Martinelli, Elisabetta Zucchi, Giulia Gianferrari, Cecilia Simonini, Annalisa Gessani, Andrea Ghezzi, and Marco Vinceti);
- Dipartimento di Scienze Biomediche e Neuromotorie, University of Bologna, and IRCCS Istituto delle Scienze Neurologiche di Bologna, Bellaria Hospital, Bologna, Italy (Veria Vacchiano, Luigi Bonan, Silvia De Pasqua and Rocco Liguori);
- IRCCS Istituto delle Scienze Neurologiche di Bologna, Department of Neurology and Stroke Center, Maggiore Hospital, Bologna, Italy (Anna Maria Borghi and Andrea Zini);
- IRCCS Istituto delle Scienze Neurologiche di Bologna, UOC Interaziendale Clinica Neurologica Metropolitana (NeuroMet), Bologna, Italy (Rita Rinaldi and Maria Guarino);
- Department of Neurosciences and Rehabilitation, UOC Neurologia Provinciale, St Anna Hospital, Ferrara, Italy (Elisabetta Sette, Riccardo De Gennaro and Daniela Gragnaniello);
- Department of Neuroscience and Rehabilitation, University of Ferrara, Ferrara, Italy (Maura Pugliatti);
- Department of Neurology, IRCCS Arcispedale Santa Maria Nuova, Reggio Emilia, Italy (Elena Canali, Luca Codeluppi, and Franco Valzania);
- Department of General and Specialized Medicine, University Hospital of Parma, Parma, Italy (Lucia Zinno, Filippo Stragliati, Pietro Anceschi, Andi Nuredini, Sonia Romano, Alessandro D’Orsi, Giorgia Libelli);
- Department of Neurology, Fidenza Hospital, Parma, Italy (Doriana Medici and Giovanna Pilurzi);
- Department of Neurology, G. Da Saliceto Hospital, Piacenza, Italy (Emilio Terlizzi and Paolo Immovilli);
- Department of Neurology, Carpi Hospital, Modena, Italy (Mario Santangelo);
- Department of Neurology, Imola Hospital, Bologna, Italy (Patrizia De Massis);
- Department of Neurology, Faenza and Ravenna Hospital, Ravenna, Italy (Matteo Gizzi, Marco Currò Dossi, Pietro Querzani and Maria Grazia Piscaglia);
- Department of Neurology, Bufalini Hospital, Cesena, Italy (Simonetta Morresi, Maria Vitiello, and Marco Longoni);
- Department of Neurology, Forlì Hospital, Forlì, Italy (Alberto Patuelli, Susanna Malagù, Francesca Bianchi, and Marco Longoni);
- Department of Neurology, Infermi Hospital, Rimini, Italy (Cristiana Ganino, Tommaso Baldini and Claudio Callegarini);
- Department of Hospital Services, Emilia Romagna Regional Health Authority, Bologna, Italy (Salvatore Ferro).

**Table S3** Members of the ERRALS Group.

**Fig. S1** Distribution of between-patient and within-patient variability in CSF measures, quantified as (i) the deviation between each patient’s mean value and the overall cohort mean, and (ii) the deviation between longitudinal measurements and their corresponding patient-specific mean (for patients with two observations), respectively.**Fig. S2** Repeated 5-fold CV (blue) and training (black) performance of the glmnet fitted multivariate Cox models at different values of the regularization parameter λ. For the baseline Cox model (left), the selected model comprises 11 covariates, associated with a CV C-index of 0.68 (whole-dataset C-index=0.79). For the longitudinal Cox model (right), the time-dependent version of the C-index was employed, achieving a mean CV value of 0.68 and leading to the selection of a multivariate model including 7 predictors (whole-dataset C^td^-index = 0.75). The plots show the mean values and standard error ranges (shaded area) over 50 iterations. The number of variables included in the multivariate model at different values of the regularization parameter λ is also shown.

**Fig. S3** Kaplan-Meier survival estimates of RAP-C9 versus CTRL-C9, after filtering for correcting sampling-related biases. No significant difference between the two groups has been detected via log-rank test (p=0.44).

**Fig. S4** Plot of scaled Schoenfield residuals. The estimated Cox regression coefficient versus time (solid line) suggests time-dependent dynamics that are coherent with treatment administration timings (p = 0.06).

**Fig. S5** Comparison of baseline ALSFRS-R score distribution within cohorts. The RAP-C9 cohort showed a higher mean baseline ALSFRS-R score (41.0, n=6) than both the non-RAP-C9 participants within RAP-ALS (38.4, n=57; Welch’s t-test p=0.048) and the CTRL-C9 cohort (38.8, n=25; p=0.156). Only 25 out of 40 CTRL-C9 patients are considered due to substantial missingness in ALSFRS-R measures. Black dots indicate mean values.
